# Investigating cerebral neurovascular responses to hyperglycemia in a rat model of type 2 diabetes using multimodal assessment techniques

**DOI:** 10.1016/j.isci.2024.110108

**Published:** 2024-05-24

**Authors:** Shaoyu Yen, Yuhling Wang, Lun-De Liao

**Affiliations:** 1 Institute of Biomedical Engineering and Nanomedicine, National Health Research Institutes, 35, Keyan Road, Zhunan Town, Miaoli County 350, Taiwan; 2 Department of Electrical Engineering, National United University, No. 2, Lianda, Nanshili, Miaoli City 36063, Taiwan

**Keywords:** physiology, molecular biology, neuroscience, techniques in neuroscience

## Abstract

To study neurovascular function in type 2 diabetes mellitus (T2DM), we established a high-fat diet/streptozotocin (HFD/STZ) rat model. Electrocorticography-laser speckle contrast imaging (ECoG-LSCI) revealed that the somatosensory-evoked potential (SSEP) amplitude and blood perfusion volume were significantly lower in the HFD/STZ group. Cortical spreading depression (CSD) velocity was used as a measure of neurovascular function, and the results showed that the blood flow velocity and the number of CSD events were significantly lower in the HFD/STZ group. In addition, to compare changes during acute hyperglycemia and hyperglycemia, we used intraperitoneal injection (IPI) of glucose to induce transient hyperglycemia. The results showed that CSD velocity and blood flow were significantly reduced in the IPI group. The significant neurovascular changes observed in the brains of rats in the HFD/STZ group suggest that changes in neuronal apoptosis may play a role in altered glucose homeostasis in T2DM.

## Highlights

• ECoG-LSCI observes the effect of hyperglycemia on cerebral neurovascular function
• Acute hyperglycemia leads to reduced blood perfusion and reduced CSD velocity
• With T2DM, blood perfusion, CSD speed, and CSD number decrease
• T2DM impairs cerebrovascular function and extracellular ion concentration buffering

## Graphical abstract

**Figure:**
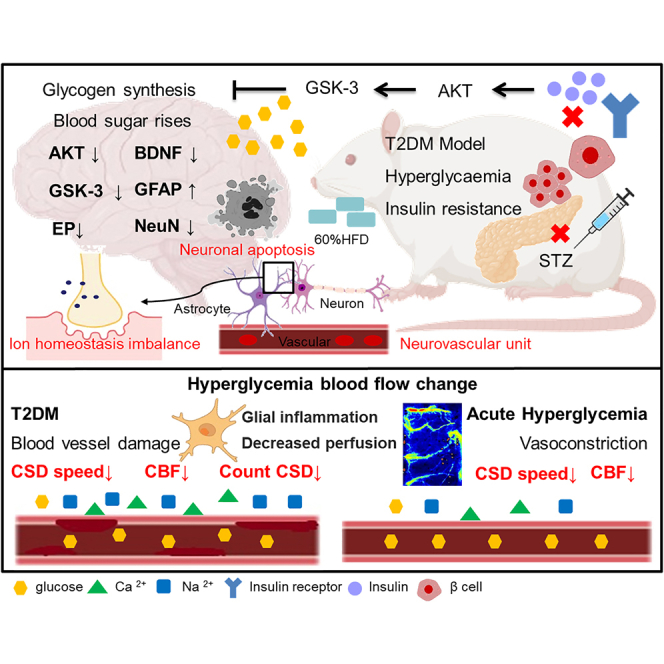

## Introduction

Diabetes is a chronic endocrine disease characterized by abnormalities in insulin synthesis or action in peripheral tissues, causing metabolic changes and hyperglycemia, leading to various complications, such as diabetic nephropathy,^1^ gestational diabetes mellitus (GDM),^2^ stroke,^3^ and diabetic foot ulcer (DFU).^4^ The incidence of obesity and type 2 diabetes (T2DM) is increasing globally, resulting in increasing clinical, social, and economic health burdens.^5^^,^^6^ The interplay between unhealthy eating habits, obesity, and diabetes has a clear impact. Obesity is a key risk factor for the development of insulin resistance and T2DM. A high-fat diet weakens the intestinal barrier and gut microbes.^7^ It is closely associated with the development of diabetes, making it a potential target for diabetes treatment. The gut-brain axis is considered the main signaling pathway controlling glucose homeostasis.^8^^,^^9^ The gut-brain axis is a bidirectional hormonal and neural signaling pathway that connects the gut and brain. In the regulation of metabolic homeostasis, this axis provides information to the brain not only through the nervous system but also through the continuous flow of microbial, endocrine, metabolic, and immune information. People with prediabetes have impaired fasting glucose levels, poor glucose tolerance, or both, which are frequently accompanied by insulin resistance. Insulin resistance develops early in T2DM, when β cell degeneration and relative insulin shortage limit glucose tolerance. The transition from a metabolically healthy to a prediabetic state is frequently accompanied by obesity, which is characterized by hyperinsulinemia, insulin resistance, and dyslipidemia. Insulin resistance affects both peripherally and centrally. At the peripheral level, a lack of response in the liver, skeletal muscle, and adipose tissue to the hormones released following an increase in blood glucose is observed. At the central level, disturbances in mitochondrial function and cellular insulin signaling in the brain are observed. In particular, the phosphoinositide 3-kinase–protein kinase B/Akt  (PI3K-PKB/Akt) pathway affects cell survival, energy metabolism, synaptic plasticity, and memory and learning processes.^10^ The high-fat diet/streptozotocin (HFD/STZ) model of T2DM exhibits insulin resistance that is remarkably comparable to that in humans with T2DM.^11^

Insulin receptors are expressed throughout the brain, with higher levels in metabolically active regions such as the hippocampus, cerebral cortex, hypothalamus, and cerebellum, indicating an important role for insulin in the central nervous system.^12^^,^^13^ Neuronal survival, synaptic plasticity, memory, learning, cell proliferation, development, and glucose homeostasis are all associated with insulin. Insulin signaling pathway proteins are crucial for these functions. Glucose metabolism is regulated by Akt, also known as protein kinase B. By translocating glucose transporter type 4 (GLUT4) to the plasma membrane, Akt regulates the biological functions of insulin, facilitating glucose uptake and phosphorylation. It also inhibits glycogen synthase kinase-3 (GSK3) activity, thereby increasing active glycogen synthase levels and promoting glycogen synthesis.^14^^,^^15^^,^^16^

Dysfunction in the brain may result from disturbances in neuronal glucose transport and metabolism, increased free radical production, or decreased brain-derived neurotrophic factor (BDNF) production during hyperglycemia, as the brain is primarily dependent on glucose. BDNF relies upon neurons for survival, growth, synapse formation and function, and the ability to reduce high blood sugar. Common features of Alzheimer’s disease include systemic insulin resistance and defects in insulin signaling in the brain, making T2DM an important risk factor for Alzheimer’s disease. Astrocytic hyperplasia and high expression of the astrocyte activation marker protein glial fibrillary acidic protein (GFAP) are caused by systemic insulin resistance and sensory neuropathy. Neuroinflammation resulting from neurodegeneration is indicated by increased GFAP expression, which leads to cognitive impairment. Metabolic homeostasis becomes impaired and dysregulated, which is associated with reduced sensory nerve conduction velocity, thermal hypoalgesia, and a decrease in nerve fiber density within the epidermis of the hind paw skin.^17^^,^^18^

Diabetes is a metabolic disease that is complex in nature and characterized by hyperglycemia, leading to macrovascular and microvascular complications. Regional cerebral blood flow (CBF) (rCBF) can be used as a measure of neurovascular function; changes in rCBF are driven by neural activity in the same region, and reduced CBF and cerebrovascular reactivity are associated with T2DM. The unifying mechanism of diabetic tissue damage in the brain is hyperglycemia because glucose must cross the blood-brain barrier (BBB) and be metabolized to provide energy. Hyperglycemia within endothelial and neuronal cells is caused by altered regulation of glucose transporters. The rCBF is affected during nerve stimulation by various vasoactive substances. Local changes in neuronal activity can be reliably monitored by measuring hemodynamic changes, such as changes in CBF, which is used as a measure of neurovascular function. Optical imaging techniques such as laser speckle contrast imaging (LSCI) and functional magnetic resonance imaging (fMRI) are necessary for detecting and analyzing local hyperemic changes.^19^ Changes in cortical CBF can be visualized in animals using LSCI, and the animals can be imaged while awake and active, enabling unresolved questions about brain function to be addressed.^20^ Another technique for assessing neurophysiology is electroencephalography (EEG) and electrocorticography (ECoG), two important neurophysiological techniques used to monitor and map electrical activity in the brain. Noninvasive EEG has been used to study brain disease and dynamic decision-making in human cognitive neuroscience, and EEG technology has been used to study behavioral effects.^21^^,^^22^^,^^23^ ECoG is an ideal method for recording real-time neural signals from specific brain regions to assess changes in somatosensory evoked potentials (SSEPs).

Neurovascular coupling (NVC) is an important mechanism in which changes in blood flow in the brain are accompanied by local neural activity. Cortical spreading depression (CSD) is a slowly propagating wave of altered brain activity involving acute changes in neuronal, glial, and vascular function. Monitoring the hemodynamic response of cerebral vessels during CSD can help elucidate the mechanisms of CSD and related neurological diseases.^24^^,^^25^ CSD is a slowly propagating (2–6 mm/min) massive neuronal depolarization wave in the cerebral cortex that causes metabolic and hemodynamic changes and is characterized by significant inhibition of neuronal activity. During CSD, normal brain tissue is affected, and CBF, glucose metabolism, and transmembrane ion transport are all altered. Therefore, understanding the dynamics of CBF during CSD is a key issue in the treatment of neurological diseases. Most tools used to monitor CSD, such as diffuse optical imaging (DOI) and LSCI, can capture CBF changes. Additionally, it is important to examine neurovascular function in the brain. ECoG is an ideal method for recording immediate neural signals from specific brain regions to assess changes in SSEPs. Our laboratory can simultaneously perform ECoG LSCI (hereinafter referred to as ECoG-LSCI) to record and comprehensively evaluate neurovascular function.^26^

Many studies have proven that diabetes affects the brain by decreasing blood sugar metabolism, leading to abnormalities. In this study, we used two hyperglycemia animal models to compare changes in brain function and comprehensively evaluated neurovascular function using ECoG integrated with LSCI. ECoG was used to record the response of the forepaw to electrical stimulation while observing blood flow changes, 4 M KCl was used to induce CSD as an indicator of the neurovascular response, and LSCI was used to measure blood flow to evaluate postoperative CBF changes. ECoG-LSCI is a method for simultaneously assessing neurovascular responses. We used western blotting to analyze changes in the levels of neurotrophic factor and insulin regulation markers in the brains of control and STZ/HFD model rats.

## Results

### Changes in body weight and blood glucose levels in HFD/STZ model rats

**Figure 1:**
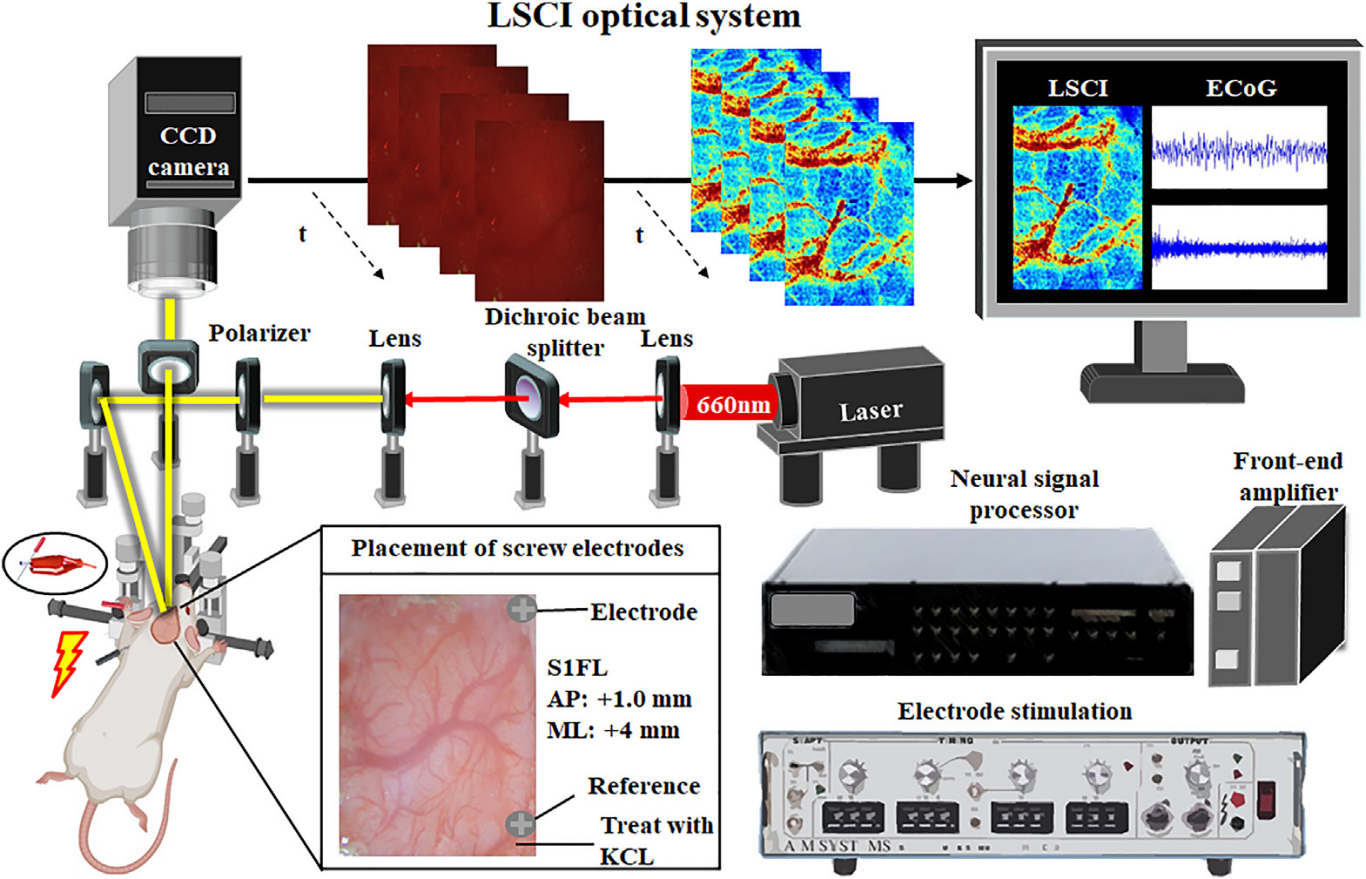
ECoG-LSCI setup and experimental protocol To assess ECoG signals and cerebral blood flow, an ECoG-LSCI neurovascular imaging system was utilized. Screw electrodes implanted in the S1FL were used to record neural activity via the ECoG. Electrical shocks were applied to the forepaws to assess changes in brain wave patterns in response to electrical stimulation. Concurrently, following craniotomy, an LSCI system was used to irradiate the cortex with a 660 nm laser. After images were taken with a CCD camera and uploaded to a computer, we used MATLAB for cerebral blood flow analysis. An ECoG recording from the S1FL (AP = +1.0 mm, ML = +4.0 mm), the reference electrode location, and the location where KCl was administered, along with the skull and the screw electrode placement, are shown in the picture.

**Figure 2:**
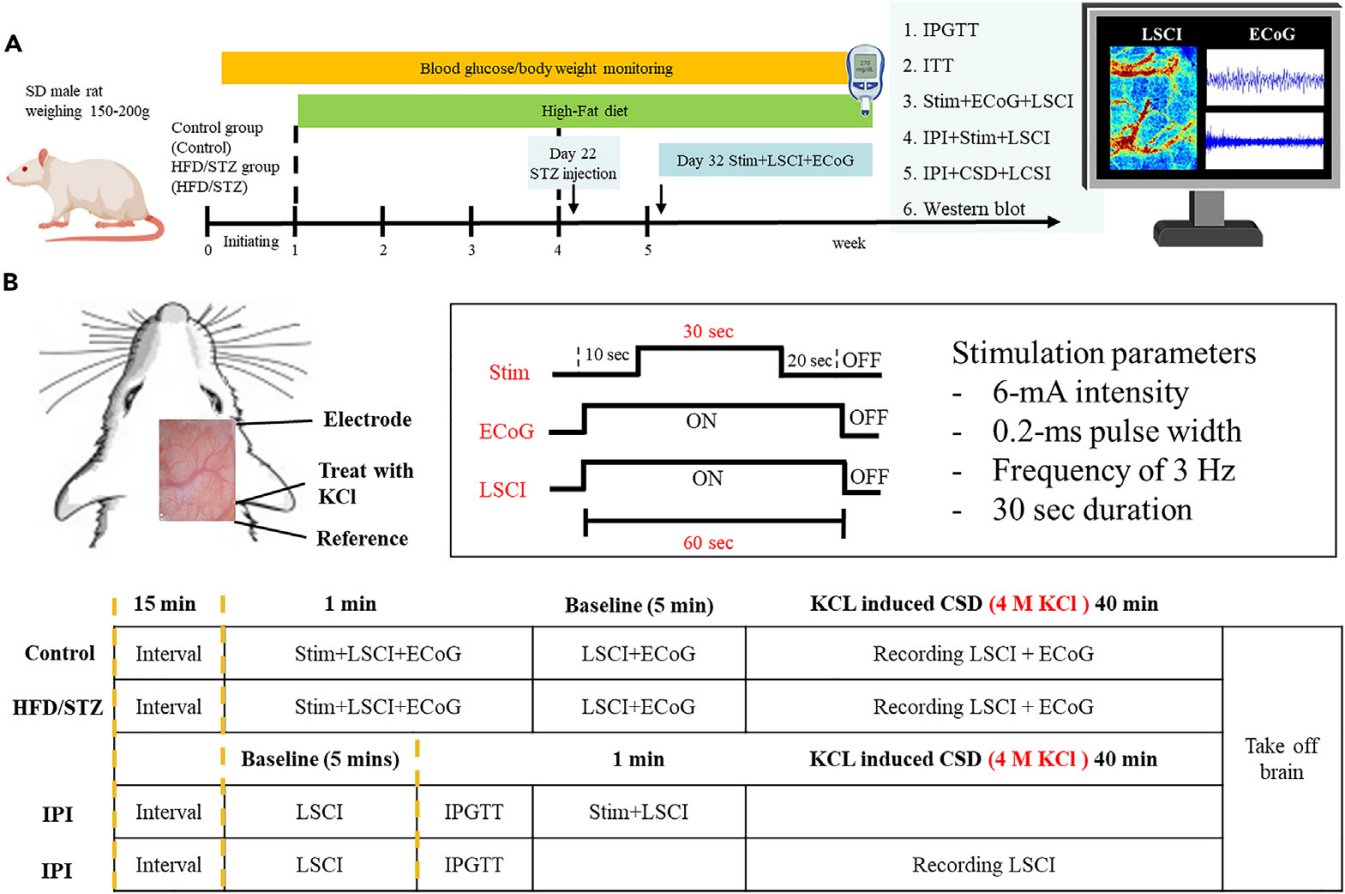
Experimental schedule for the establishment of the HFD/STZ-induced T2DM animal model and ECoG-LSCI during peripheral stimulation (A) Rats were divided into two groups: the control and HFD/STZ groups. On day 21, the rats were administered STZ (40 mg/kg/d × 5 days, intraperitoneal injection) for 5 consecutive days, and blood glucose levels and body weights were monitored once a week until day 49. Diabetic rats (defined as those with blood glucose levels ≥ 288 mg/dL) were identified 28 days after STZ administration. Next, a glucose metabolism test was performed. After ECoG-LSCI, the rats were sacrificed, and the brains were removed for protein expression analysis. (B) One-half of the rat brain was dissected, and the location of the S1FL was determined. There were 3 holes in the brain tissue: 1 for the ECoG electrode, 1 for the reference electrode, and 1 for KCl delivery. Electrical signals in the S1FL were detected after 30 s of electrical stimulation at 6 mA. A combination of electrophysiology and LSCI was used to monitor electrical stimulation-evoked activity in the S1FL after 3 stimuli (30 s of electrical stimulation at 6 mA with 60-s intervals). ECoG and LSCI were used to measure S1FL activity in response to electrical stimulation. Moreover, 4 M KCl was used to induce CSD, and CSD data, including cerebral blood flow changes, were collected for 40 min. Blood flow changes during electrical stimulation were recorded after acute hyperglycemia was induced by IPI. After IPI-induced acute hyperglycemia, KCl was applied, and blood flow changes were recorded.

**Figure 3:**
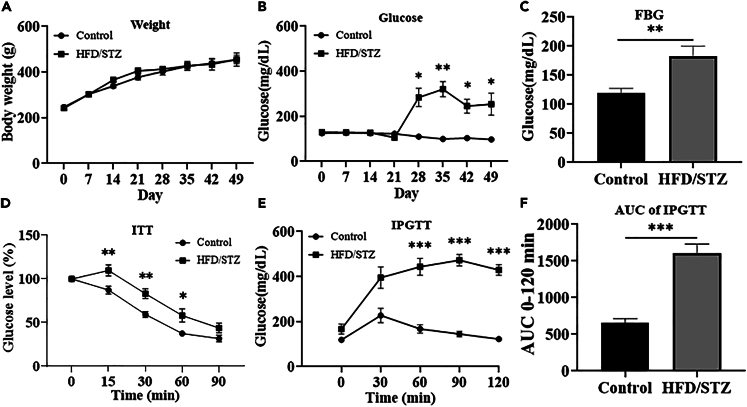
Changes in body weight and blood glucose levels in HFD/STZ-induced diabetic model rats (A) Body weight changes in control rats and HFD/STZ-induced diabetic model rats were not significant. (B) After STZ injection, the blood glucose concentration in the HFD/STZ group was significantly greater than that in the control group on day 28. (C) Fasting blood glucose levels in the control group and HFD/STZ group. The fasting blood glucose level in the HFD/STZ group was significantly greater than that in the control group. (D) According to the insulin tolerance test, HFD/STZ model rats were not sensitive to insulin and showed insulin resistance. (E) According to the glucose tolerance test, blood glucose levels in the HFD/STZ group were greater than those in the control group. (F) The area under the curve (AUC) for the glucose tolerance test was significantly greater in the HFD/STZ group than in the control group. ∗*p* < 0.05, ∗∗*p* < 0.01, ∗∗∗*p* < 0.001 (n = 8 in each group). Data are represented as mean ± SD.

To confirm the successful establishment of the hyperglycemia animal model, the body weights and blood glucose levels of the rats were measured on days 0, 7, 14, 21, 28, 35, 42, and 49. Next, to study the impact of diabetes on brain function, we used the ECoG-LSCI system (Figure 1) to evaluate the neurovascular function of the hyperglycemia animal model (Figure 2). Impaired hepatic glucose metabolism is a typical feature of T2DM. There was also no significant difference in body weight between the control and HFD/STZ groups (Figure 3A). Injection of low-dose STZ slightly impaired insulin secretion. The blood glucose level was 288 mg/dL on day 28, and this value was maintained until the end of the 49-day experiment (109.5 ± 2.1 vs. 283.6 ± 40.5 mg/dL, *p* < 0.05; Figure 3B).

### High-fat diet feeding impairs glucose tolerance and causes insulin resistance

Diabetes was induced using a high-fat diet and low-dose STZ, and the metabolic capacity of the rats was evaluated on day 32. Fasting blood glucose levels were greater in the HFD/STZ group than in the control group (*p* < 0.01; Figure 3C). In the insulin resistance experiments, HFD/STZ model rats were found to be insensitive to insulin and to exhibit insulin resistance (*p* < 0.01; Figure 3D). The intraperitoneal glucose tolerance test (IPGTT) is used to diagnose diabetes. An elevated blood sugar level indicates impaired glucose metabolism. Our results showed that the HFD/STZ group was glucose intolerant (*p* < 0.001; Figure 3E), as the mechanisms that regulate glucose concentrations in the blood were impaired. Normal rats generally have a glucose curve with a typical shape and a normal area under the curve (AUC).^27^ In comparison, the area under the glucose curve was greater for glucose-intolerant rats than for control rats (*p* < 0.001; Figure 3F).

### Effects of hyperglycemia on neurovascular function

**Figure 4:**
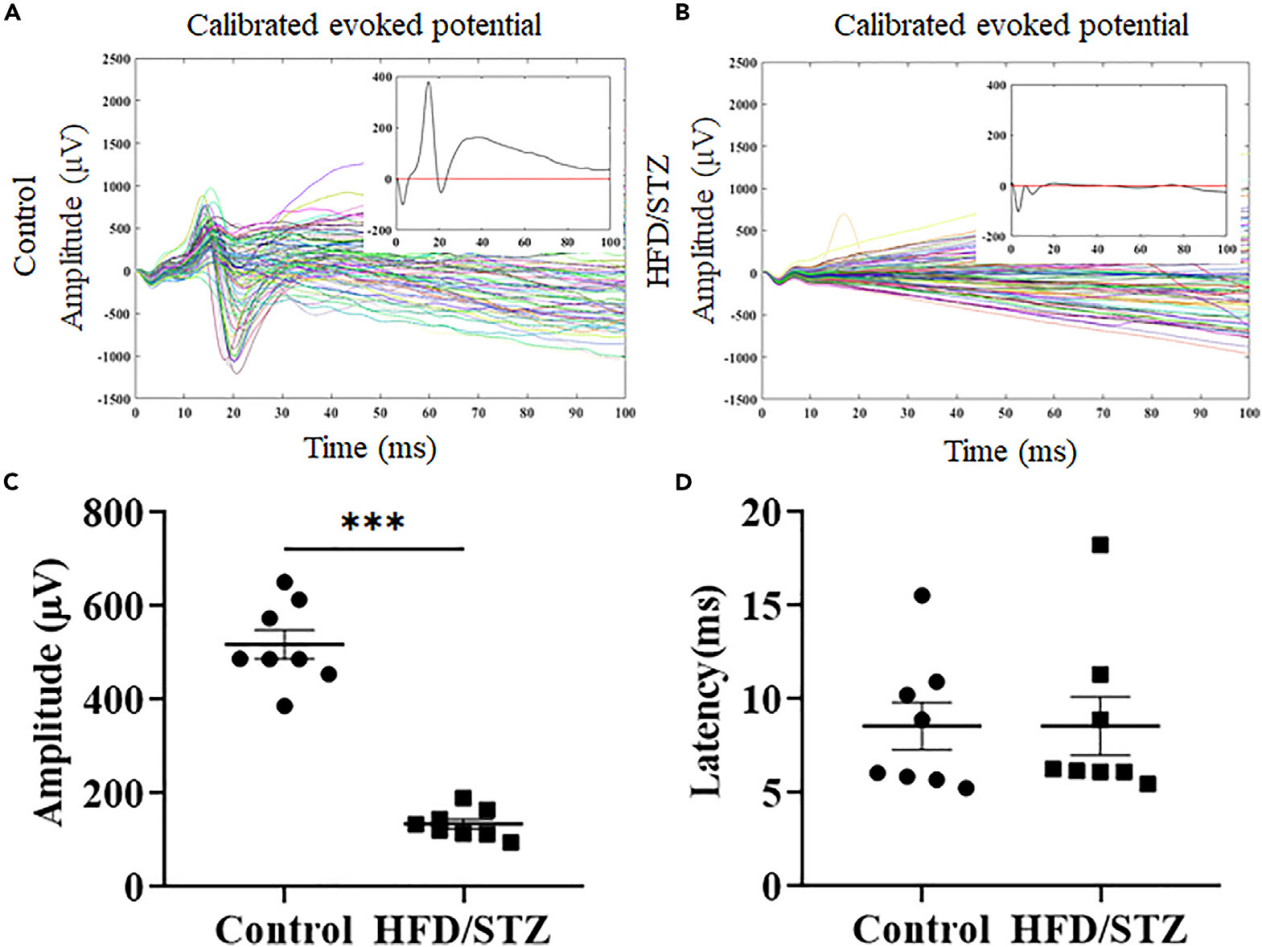
ECG recordings of rats in the control and HFD/STZ groups during electrical stimulation Three 30-s trains of electrical stimulation were applied to the S1FL. MATLAB was used to obtain calibrated evoked potentials and to analyze the average evoked potential plots. The baseline and average shock responses were determined from these plots. The electrical stimulation parameters included an amplitude of 6 mA, pulse width of 0.2 ms, frequency of 5 Hz, and duration of 30 s; the calibrated evoked potential was first determined, and then electrical stimulation was applied 10 s later for 30 s. (A) The mean evoked potential in the control group. The thumbnail represents the calibrated evoked potential. (B) Mean evoked potentials in the HFD/STZ group; the thumbnail shows the calibrated evoked potentials. (C) Analysis of electrophysiological results using MATLAB. Column analysis was performed using GraphPad Prism, and differences between the control and HFD/STZ groups were analyzed using an unpaired *t* test. Potential differences between the control and HFD/STZ groups; control vs. HFD/STZ, ∗∗∗*p* < 0.001. (D) Conduction latency; there was no significant difference between the control and HFD/STZ groups; *p* = 0.4595. (n = 8 in each group). Data are represented as mean ± SD.

**Figure 5:**
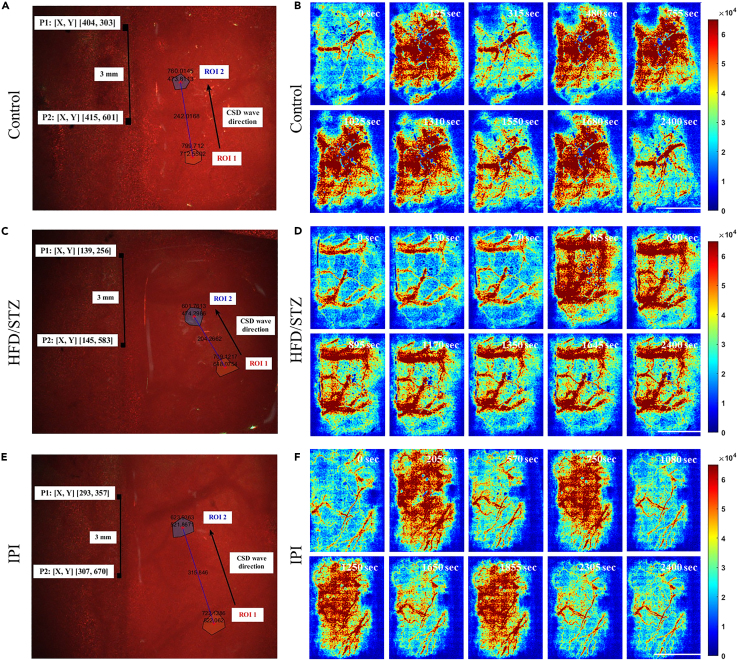
Effect of HFD/STZ-induced hyperglycemia and acute hyperglycemia on the number of CSD events and CSD velocity LSCI was used to detect alterations in cortical blood flow after KCl-induced CSD. CSD was induced by applying 4 M KCl for 40 min. The images on the left are centered on bregma, and P1 and P2 are marked. CSD velocity was determined by measuring blood flow in two circular regions of interest (ROIs) in the cerebral cortex separated by 3 mm. The velocity of the CSD was calculated by dividing the actual distance by the time difference between the peaks at that cortical location. The direction of the CSD is indicated by arrows. The degree of blood flow change over time was dependent on the distance between the two points and the time difference between the peaks at that cortical location. (A) An LSCI image of a control rat brain. (B) Dynamic changes in blood flow in the control group at 0, 125, 315, 480, 755, 1,025, 1,310, 1,550, 1,680, and 2,400 s. (C) LSCI images of the brains of rats in the HFD/STZ group. (D) Dynamic changes in blood flow in the HFD/STZ group at 0, 130, 270, 485, 690, 895, 1,170, 1,350, 1,645, and 2,400 s. (E) LSCI images of the brains of rats in the IPI group. (F) Dynamic changes in blood flow in the IPI group at 0, 205, 570, 750, 1,080, 1,250, 1,650, 1,855, 2,305, and 2,400 s. Areas of low blood perfusion are dark blue, and areas of high blood perfusion are dark red. Movies of blood flow changes are shown in (B), (D), and (F) of Videos S1, S2, and S3, respectively. Scale bar, 3 mm.

**Figure 6:**
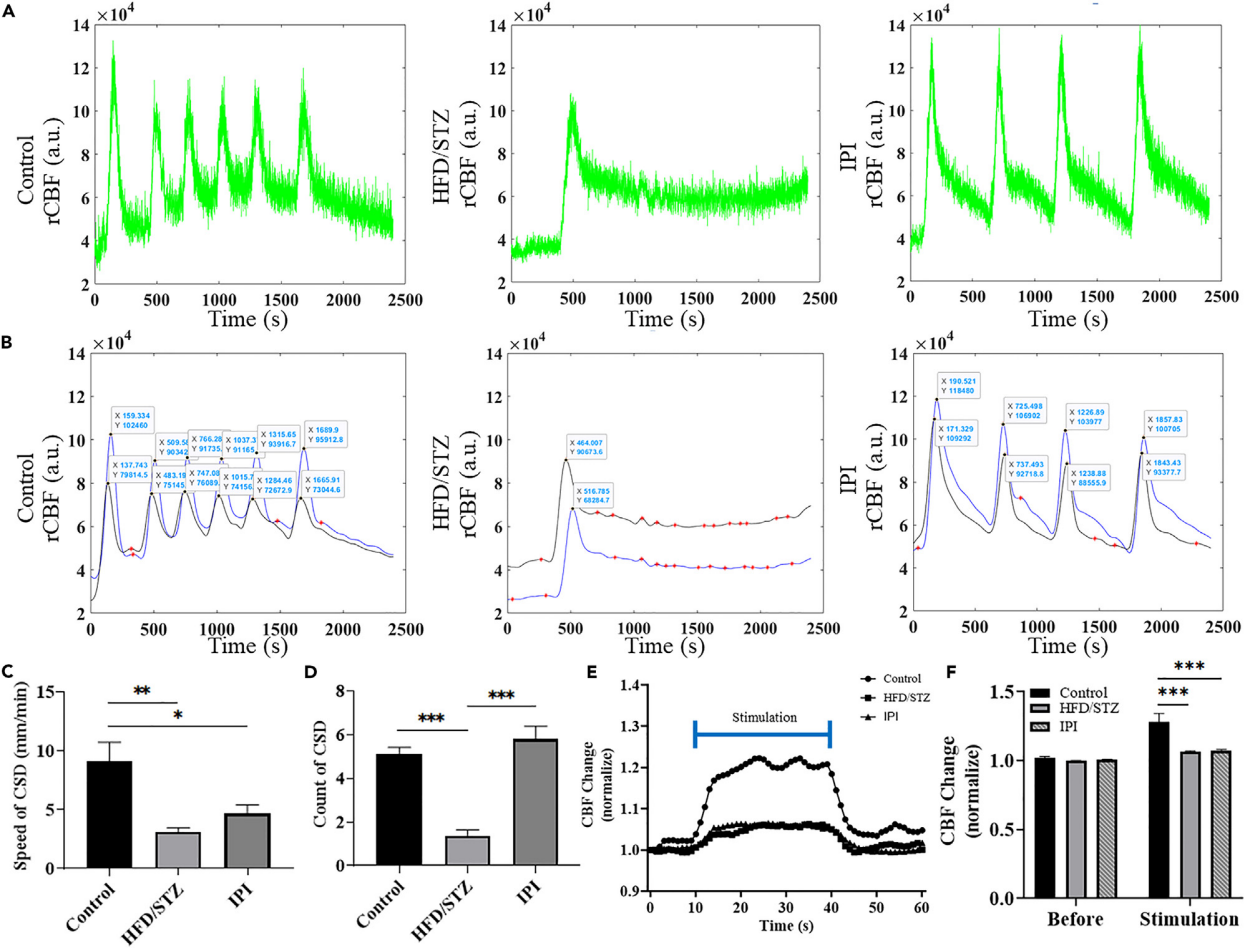
Effects of HFD/STZ and acute hyperglycemia on cerebral neurovascular coupling Cerebral blood flow changes during KCl-induced CSD were recorded by LSCI, with 2 images captured per second (frame rate 2) for a total of 2,400 s. Using MATLAB, all the images were analyzed at a rate of 2 images/second, and the values were calculated to assess the CBF and determine the conditions under which CSD occurred. (A) From left to right, cerebral blood flow analysis in the control group, HFD/STZ group, and IPI group. (B) The duration of CSD in the control group, HFD/STZ group, and IPI group (from left to right). (C) After KCl-induced CSD was analyzed using MATLAB, the difference in the number of CSD events among the control group, HFD/STZ group, and IPI group was determined. (D) Differences in CSD velocity among the control group, HFD/STZ group, and IPI group. (E) Electrical stimulation-induced changes in blood flow in the control group, HFD/STZ group, and IPI group were compared. The data were recorded for a total of 60 s. Stimulation started at 10 s and continued for 40 s. (F) There was no difference between the groups before stimulation after normalization. There was a significant difference in blood perfusion among the groups within 30 s of stimulation. Statistical analysis was performed using one-way ANOVA with GraphPad Prism. ∗*p* < 0.05, ∗∗*p* < 0.01, ∗∗∗*p* < 0.001 (n = 8 in each group). Data are represented as mean ± SD.

To study the impact of diabetes on brain function, we established a diabetic rat model. Four weeks after diabetes induction, the rats were subjected to craniotomy for ECoG-LSCI. Figures 4A and 4B show the average evoked potentials of the control and HFD/STZ groups. The thumbnail is the SSEP waveform. The SSEP amplitude in the HFD/STZ group was significantly lower than that in the control group (control group: amplitude 518.1; HFD/STZ group: amplitude 135.0, *p* < 0.001; Figure 4C). There was no significant difference in latency between the control and HFD/STZ groups (control group: latency 8.56; HFD/STZ group: latency 8.58; Figure 4D). Figures 5A, 5C, and 5E show changes in the rCBF during KCl-induced CSD in the control group, HFD/STZ group, and IPI group. Figures 5B, 5D, and 5F show the response to KCl in the control group, HFD/STZ group, and IPI group. Images of changes in the rCBF at different time points during CSD induction are shown. Blue indicates low blood perfusion, and red indicates high blood perfusion. The images in Figures 5B, 5D, and 5F (Videos S1, S2, and S3) show the rCBF distribution calculated from speckle images during CSD. Changes in the rCBF during KCl-induced CSD were evaluated. We counted the number of CSD events. There were 5 peaks in the control group during CSD, while there was 1 peak in the HFD/STZ group and 4 peaks in the IPI group. Interestingly, rCBF failed to return to baseline levels after CSD induction in the majority of the HFD/STZ model rats (Figure 6A). We also calculated the CSD velocity, and the average CSD velocity in the HFD/STZ group (3.1 mm/min) and IPI group (4.7 mm/min) was lower than that in the control group (9.1 mm/min) (Figure 6B). We also observed that the HFD/STZ group exhibited fewer CSD events, which may have been due to brain damage.

Video S1. Video of dynamic changes in blood flow in the control group (Figure 5B)

Video S2. Video of dynamic changes in blood flow in the HFD/STZ group (Figure 5D)

Video S3. Video of dynamic changes in blood flow in the IPI group (Figure 5F)

Figure 6C shows the CSD velocity. The CSD velocity in the control group was significantly greater than that in the HFD/STZ and IPI groups (*p* < 0.05). There were no significant differences between the HFD/STZ and IPI groups. Figure 6D shows the number of CSD occurrences. The number of CSD occurrences in the control group was greater than that in the HFD/STZ group, and the difference was significant (*p* < 0.001). There was also a significant difference between the HFD/STZ group and the IPI group (*p* < 0.001). However, there was no significant difference between the control group and the IPI group.

After evaluating changes in blood flow during electrical stimulation (Figure 6E), there were no differences in blood perfusion between the groups before electrical stimulation. During electrical stimulation, the blood perfusion volume in the control group was 1.28, which was significantly greater than that in the HFD/STZ group (1.06) (*p* < 0.05). The blood perfusion volume in the IPI group was 1.07 lower than that in the control group (Figure 6F).

### Effects of Akt and GSK3 phosphorylation on the brains of HFD/STZ model rats

**Figure 7:**
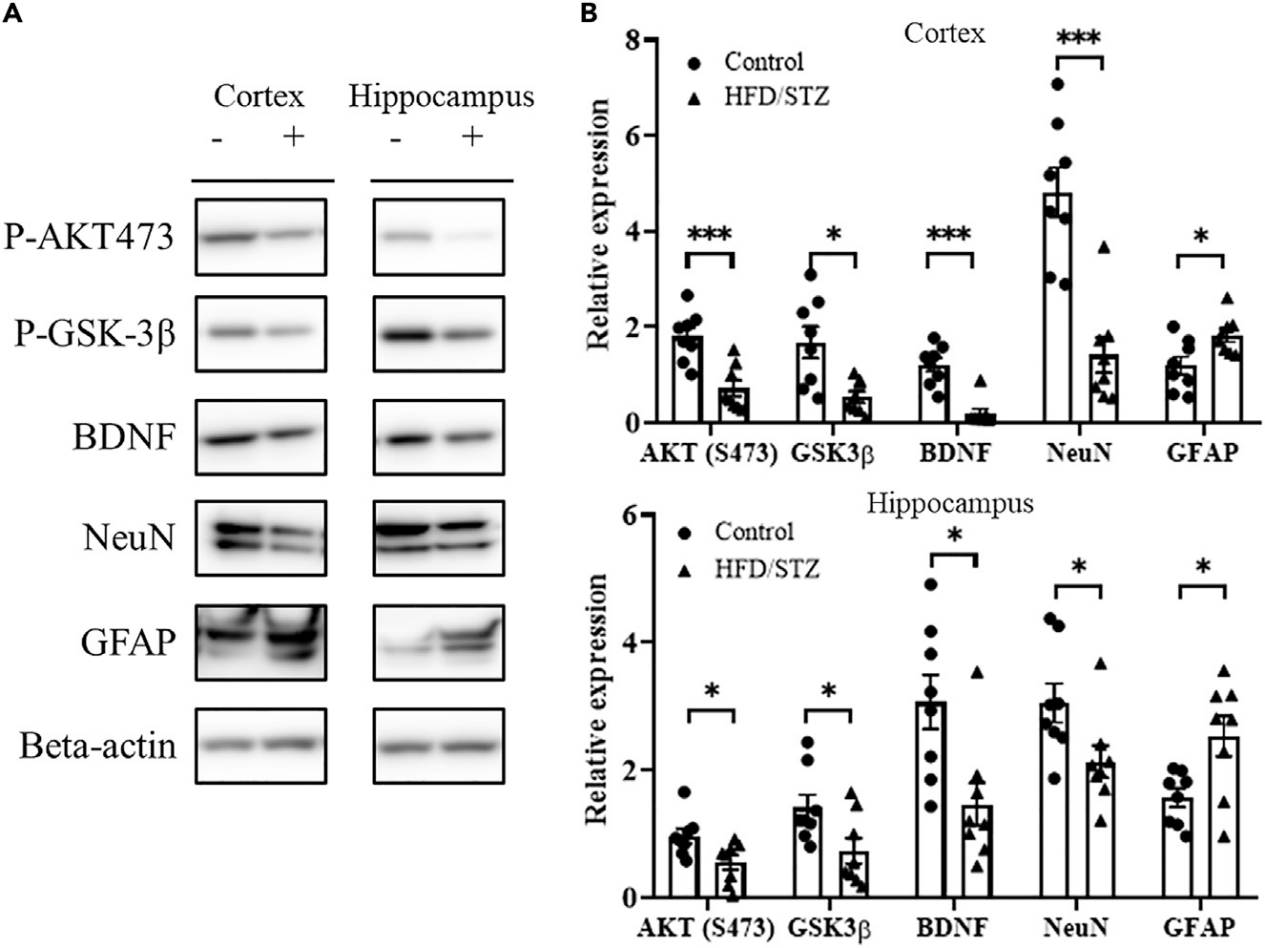
Expression levels of P-AKT473, P-GSK3β, BDNF, NeuN, and GFAP in the brains of HFD/STZ model rats (A) Representative western blot images of AKT473, GSK3β, BDNF, NeuN, GFAP, and β-actin. (B) Quantification of AKT473, GSK3β, BDNF, NeuN, GFAP, and β-actin levels. β-Actin was used as an internal control. In the western blot image, “-” indicates the control group, and “+” indicates the HFD/STZ group. Protein expression levels were quantified by densitometric analysis of the western blot bands and are presented as the means ± SEMs of eight rats per group. ∗*p* < 0.05, ∗∗∗*p* < 0.001 compared to control values. (n = 8 in each group). Data are represented as mean ± SD.

**Figure 8:**
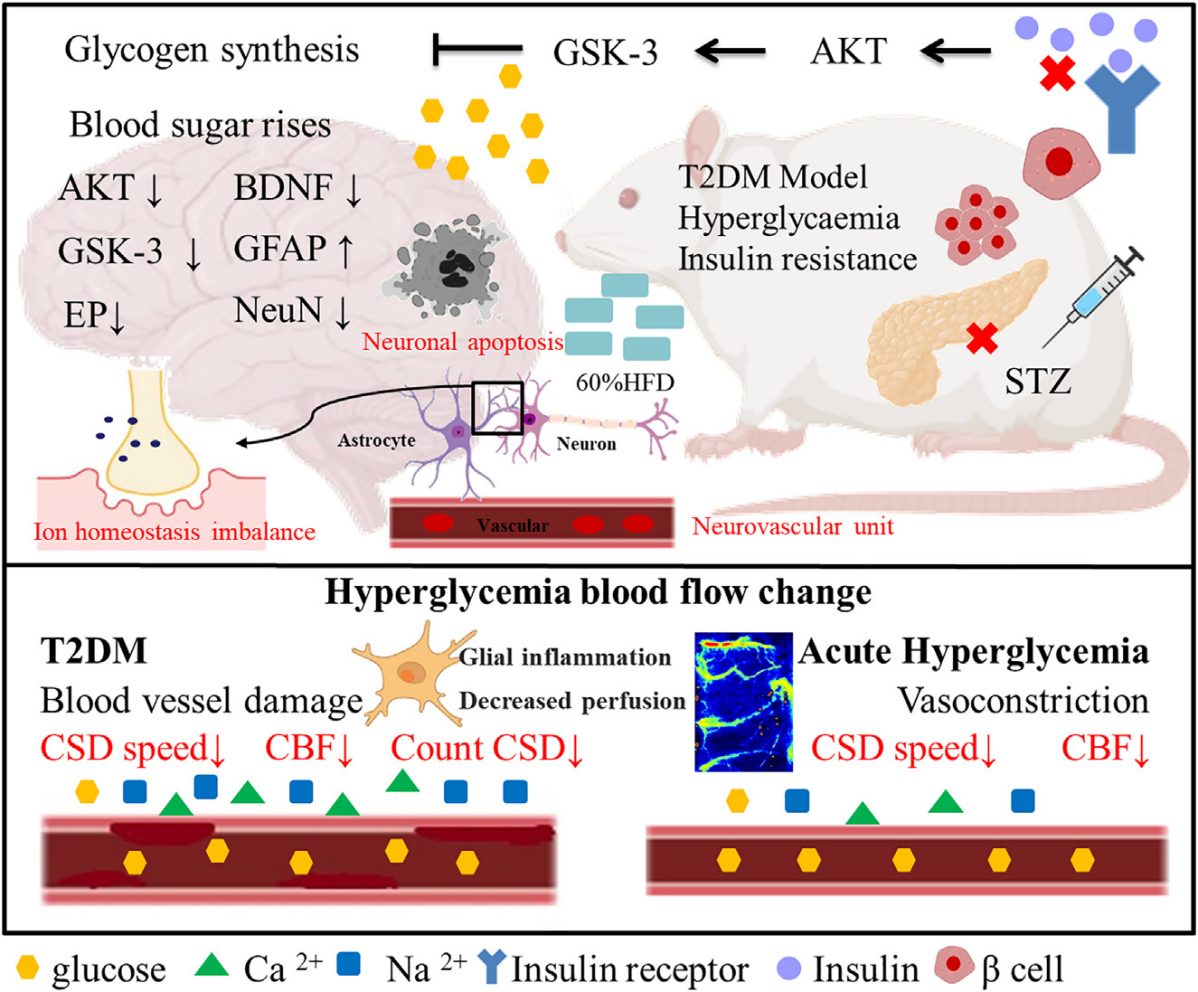
Summary of the effects of type 2 diabetes on brain signaling molecules HFD/STZ-induced diabetes results in hyperglycemia and impaired brain function. When glucose concentrations in the blood increase, tissue is damaged, resulting in a decrease in NeuN and BDNF expression and an increase in GFAP expression, causing synaptic dysfunction. Elevated glucose levels lead to decreased AKT and GSK3β levels. Dysfunction of physiological neurovascular coupling leads to decreased neuronal activity, imbalance of ion homeostasis, and abnormal blood flow regulation. NeuN, neuronal nuclear antigen; BDNF, brain-derived neurotrophic factor; AKT, AKT kinase serine; GSK3β, glycogen synthase kinase 3β.

Figures 7A and 8 show the results of western blot analysis of the rat cortex and hippocampus. Statistical analysis (Figure 7B) revealed that the protein expression of Akt in the cortex and hippocampus was significantly lower in the HFD/STZ group than in the control group (*p* < 0.05). The protein expression levels of GSK3β in the cortex and hippocampus were significantly lower in the HFD/STZ group than in the control group (*p* < 0.05). The protein expression levels of BDNF in the cortex and hippocampus were significantly lower in the HFD/STZ group than in the control group (*p* < 0.05). The protein expression level of neuronal nuclear antigen (NeuN) in the cortex and hippocampus was significantly lower in the HFD/STZ group than in the control group (*p* < 0.05). The protein expression levels of GFAP in the cortex and hippocampus were significantly greater in the HFD/STZ group than in the control group (*p* < 0.05).

## Discussion

In this study, we reviewed numerous studies using animal models of diabetes involving combinations of high-fat diets^28^^,^^29^^,^^30^^,^^31^ that, in some cases, induce hyperinsulinemia, insulin resistance,^32^^,^^33^ and glucose intolerance. Treatment with the β cell toxin STZ subsequently resulted in decreased insulin resistance in functional β cells. Although high doses of STZ severely impair insulin secretion, similar to type 1 diabetes, low doses of STZ are known to cause mild impairment of insulin secretion, consistent with T2DM. Similar to the features of the late stages of T2DM, this approach induces inflammation-mediated β cell destruction rather than the rapid β cell death induced by a single dose of STZ. This model uses a high-fat diet combined with multiple low-dose STZ injections to replicate human syndrome and metabolic characteristics.^34^^,^^35^

T2DM is caused by a relative lack of insulin production in tissues and insulin resistance. The pathophysiological process of T2DM involves the glucose metabolism pathway and insulin signaling pathway in tissues. Hyperglycemia can cause brain damage through glucose metabolism pathways. The pathogenesis of diabetes-related brain dysfunction involves multiple factors. NVC impairment caused by high blood glucose levels has been suggested as a potential mechanism. One of the major factors is that physiological changes caused by glycemic imbalance can lead to vascular endothelial damage, accompanied by altered cerebrovascular responses and thickening of the microvascular basement membrane.^36^

The neurovascular unit (NVU) is composed of a network of cerebrovascular endothelial cells (CECs), pericytes, smooth muscle cells, stellate cells, and neurons, which together regulate CBF.^37^ Reductions in NVC and cerebrovascular reactivity suggest that blood flow may reduce vessel diameter rather than just causing vessel loss. Under normal circumstances, insulin promotes glucose uptake and increases blood flow to peripheral tissues by causing vasodilation. Insulin has been shown to play a role in regulating vascular and metabolic processes. In the brain, diabetes progressively damages the vascular endothelium, making vessel walls thicker, more permeable, and less responsive to endogenous regulation of vascular tone. The NVU regulates blood supply to fulfill the energy and oxygen demands of active neurons in diabetic NVC, also known as functional hyperemia. This communication occurs between neurons, stellate cells, and cerebral blood arteries. Changes in tissue metabolism and blood flow are linked to CSD, which is represented by self-propagating waves of membrane/tissue depolarization. CSD can occur in both the healthy brain and the damaged brain; CSD occurs in both healthy and sick people when the change in response to energy demands is normal or decreased (via the upstream blood supply). Hyperglycemia may counteract the effects of increased extracellular potassium concentrations, thereby inhibiting the onset and propagation of CSD. The concept of NVC describes a cellular mechanism by which neuronal activation induces a simultaneous local increase in CBF. Increased local blood supply is critical for brain function, and damaged NVC may play an early role in triggering cognitive dysfunction in T2DM patients. Diabetes leads to neurovascular decoupling and disruption of the BBB. CECs are components of the BBB and NVU. CECs are critical in NVC, where they help regulate CBF in response to regional increases in cellular demand in the NVU. In hyperglycemia, increased energy stores delay disruption of the transmembrane ion gradient, thereby increasing the latency to CSD and reducing the CSD propagation rate. These changes resulted in reduced CBF velocity and fewer CSD episodes in the HFD/STZ group. High blood sugar levels can impair blood flow regulation. Therefore, the blood perfusion volume in the HFD/STZ group in response to electrical stimulation was lower than that in the control group. Global decreases in CBF and neurovascular uncoupling are hallmarks of normal aging and disease-related neurodegeneration. CEC dysfunction alone is sufficient to promote BBB disruption and neurovascular uncoupling. In addition to causing BBB defects, CEC dysfunction can lead to dysregulation of NVC.

T2DM is a metabolic disease that affects brain structure and function through glucotoxicity, vascular damage, brain insulin resistance, synaptic damage, neuroinflammation, and gliogenesis. Glucose can cross the BBB through insulin-related mechanisms, especially when serum concentrations are high, and the BBB cannot completely prevent peripheral glucose from reaching the brain. Therefore, when sustained hyperglycemia causes inflammation, the BBB may further weaken, leading to neurotoxicity and triggering mechanisms detrimental to neuronal survival. Therefore, we examined changes in AKT, GSK3β, NeuN, BDNF, and GFAP expression in a rat model, and our data showed that AKT and downstream GSK3β expression was reduced in the brains of rats with HFD/STZ-induced hyperglycemia. The serine/threonine kinase Akt, a downstream target of phosphoinositide 3-kinase (PI3K), is transiently phosphorylated at Ser473, and the level of the phosphorylated form of the downstream signaling molecule GSK3β is significantly reduced. These results suggest dysregulation of the PI3K-Akt signaling pathway and inhibition of *p*-GSK3β.^38^ The significant changes in brain insulin signaling expression and cell survival pathways observed in the brains of diabetic animals imply that alterations in neuronal apoptosis may play a role in the alterations in glucose homeostasis observed in T2DM patients. Our results also revealed that hyperglycemia-induced neuroinflammation resulted in decreased NeuN and BDNF expression and abnormally increased GFAP expression in the HFD/STZ group. Some studies have suggested that diabetic rats exhibit a massive loss of synaptic proteins, neuronal apoptosis, synapse loss, and impaired electrophysiology. Alterations in the function of synaptic neurotransmitters and changes in their activity may disrupt the normal functioning of the nervous system.^39^^,^^40^ Hyperglycemia may directly cause neuronal damage, and, in rats, hyperglycemia (streptozotocin-induced diabetes) was found to cause structural changes in neurons and impaired memory.^41^ In this study, we used ECoG-LSCI, electrical stimulation, and KCl-induced CSD to evaluate the effects of diabetes on brain function. Because these methods require exposure of the brain surface, they can only be used to observe neuronal function in limited areas of the brain. However, the effects of diabetes on the brain are widespread. In the future, we will validate our results using fMRI or other measures that more directly reflect neuronal reactivity.

Our results showed that rats with acute hyperglycemia had a lower blood perfusion volume and slower CSD velocity than the control rats. However, we discovered an interesting phenomenon. There was no significant difference in the number of CSD events between the acute hyperglycemia group and the control group. The literature indicates that hyperglycemia is related to the impairment of vascular endothelial function, resulting in decreased vasodilation and changes in blood flow that may lead to ischemia.^42^ Therefore, chronic hyperglycemia may be caused by damaged blood vessels and thickened blood vessel walls, resulting in slower blood flow, while acute hyperglycemia impairs vasoconstriction while sparing ion channels and neurotransmission. Long-term hyperglycemia can cause progressive functional damage to brain neurons. Changes in blood sugar status in diabetic patients are prodromal symptoms of BBB damage. The increased incidence of hemorrhagic transformation in hyperglycemic patients after stroke also suggests possible disruption of BBB integrity.^43^ T2DM may involve the cortex in multiple brain regions and is associated with reduced regional cerebral perfusion and vascular reactivity, and reduced blood flow may reflect the combined effects of microvascular disease and metabolic tissue damage. These findings are clinically relevant to functional outcomes such as cognition and balance in older patients with diabetes. The mechanisms by which hypoperfusion is associated with neurodegeneration and functional outcomes in T2DM patients warrant further investigation.^44^^,^^45^

### Limitations of the study

In this study, we used ECoG-LSCI, electrical stimulation, and KCl-induced CSD to evaluate the effects of diabetes on brain function. Because these methods require exposure of the brain surface, they can only be used to observe neuronal function in limited areas of the brain. However, the effects of diabetes on the brain are widespread. In the future, we should validate our results using fMRI or other measures that more directly reflect neuronal reactivity. We are currently using western blotting to verify, according to different brain regions, that diabetes has different effects on protein levels in different brain regions. Therefore, future studies are needed to explore the functional roles of these regional tissues.

## STAR★Methods

### Key resources table

**Table:** 

REAGENT or RESOURCE	SOURCE	IDENTIFIER
**Antibodies**		

Rabbit monoclonal anti-BDNF	Abcam	Cat# ab108319; RRID:AB_10862052
Rabbit monoclonal anti-NeuN	Abcam	Cat# ab177487; RRID:AB_2532109
Phospho-Akt (Ser473)	Cell Signaling Technology	Cat# 9271; RRID:AB_329825
Goat polyclonal anti-GFAP	Santa Cruz	Cat# sc-6170; RRID:AB_641021
Mouse monoclonal anti-actin	Santa Cruz	Cat# sc-47778; RRID:AB_626632
Phospho-GSK-3β (Ser9)	Cell Signaling Technology	Cat# 9336; RRID:AB_331405
Goat Anti-Rabbit IgG - H&L Polyclonal antibody, Hrp Conjugated	Abcam	Cat# ab6721; RRID:AB_955447
Goat polyclonal Secondary Antibody to Mouse IgG - H&L	Abcam	Cat# ab6789; RRID:AB_955439

**Chemicals, peptides, and recombinant proteins**		

Streptozocin	Sigma-Aldrich	Cat# PPC2020
RIPA buffer	Visual Protein	Cat# RP05-100
Protease and Phosphatase inhibitor	Sigma-Aldrich	Cat# PPC2020

**Critical commercial assays**		

BCA protein assay kit	ThermoFisher	Cat# 23225, 23227
Immobilon Western Chemiluminescent HRP Substrate	Millipore	WBKLS0500

**Experimental models: Organisms/strains**		

Rat: Sprague–Dawley	LASCO	N/A

**Software and algorithms**		

ImageJ	ImageJ	https://imagej.nih.gov/ij/index.html
GraphPad Prism v.8.0.1	GraphPad	https://www.graphpad.com/scientific-software/prism

### Resource availability

#### Lead contact

Further information and requests for resources and reagents should be directed to and will be fulfilled by the lead contact, Lun-De Liao (ldliao@nhri.edu.tw).

#### Materials availability

This study did not generate new unique reagents.

#### Data and code availability

• Original western blot images reported in this paper will be shared by the lead contact upon request.
• All original code will be shared by the lead contact upon request.
• Any addition information required to reanalyze the data reported in this paper is available from the lead contact Lun-De Liao (ldliao@nhri.edu.tw) upon request.

### Experimental model and study participant details

#### Animals

The animals were housed in a controlled environment on a 12-h dark/light cycle at a constant temperature and humidity. Food and water were provided *ad libitum*. A total of 32 adult male Sprague‒Dawley (SD) rats (LASCO, Taipei, Taiwan), weighing 150 to 200 g (8–10 weeks old), were used and equally divided into two groups: the control group (*n* = 24) and the HDF/STZ group (*n* = 8). Rat health, locomotion, coat condition, body weight stability, and food and water consumption were monitored weekly. Given that diabetic rats drink a large amount of water, water and food were replenished regularly, and the cage was cleaned frequently. The body weights and blood glucose levels of the rats were measured weekly. All animal experiments were performed in accordance with the Institutional Animal Care and Use Committee (IACUC) guidelines of the National Health Research Institute (NHRI), Taiwan (approved protocol number: NHRI-IACUC-112004-A).

#### Procedures for diabetes induction

A diabetic rat model was created by administering moderate doses of STZ to animals previously fed a high-fat diet to induce insulin resistance. This process results in hyperglycemia associated with hyperinsulinemia and insulin resistance.^34^ The body weight of each rat was recorded weekly. Blood glucose levels were measured every 7 days using an Accu-Chek blood glucose meter (Roche, Germany). Control animals received standard rodent chow, and T2DM model animals received a high-fat diet (D12492 diet; Research Diets, http://www.researchdiets.com/opensource-diets/diet-induced-disease-models/obesity) for 21 days. On day 22, the animals in the HFD/STZ group received a single dose of STZ (40 mg/kg body weight) intraperitoneally and were fasted for 8 h. The control animals received vehicle only (50 mM citrate buffer, pH 4.5). After the induction of hyperglycemia over a period of 10 days, blood glucose levels were measured to confirm the development of diabetes.^46^ Animals were considered diabetic if their postprandial blood glucose levels were greater than or equal to 288 mg/dL (Figure 2A). As diabetes is associated with polyuria, frequent bedding changes are needed.

### Method details

#### Electrocorticography–laser speckle contrast imaging (ECoG–LSCI) system

In this study, we used a combination of ECoG and LSCI, as shown in Figure 1, to examine the effects of T2DM on cerebral neurovascular function. The ECoG–LSCI system allowed simultaneous measurement of both rCBF and neuronal activity. To illuminate the region of interest, a 660 nm laser (100 mW; RM-CW04-100, Unice E-O Service, Inc., located in Taoyuan, Taiwan) was used. The laser beam was expanded through a plano-convex lens (75 mm; LA1608-A; Thorlabs, Inc., in Newton, NJ, USA) to a size of approximately 40 × 30 mm to properly illuminate the exposed area of the cortex. The illuminated area was imaged by a 16-bit charge-coupled device (CCD) camera (4.65 × 4.65 μm pixels; DR2-08S2M/C-EX-CS, Point Gray Research, Inc., in Richmond, BC, Canada) using an adjustable magnification lens (0.3–1 ×, maximum f/4.5) with a 2× extender. To eliminate scattering, a linear polarizer was positioned in front of the CCD image collection lens at a working distance of approximately 5 cm. Laser speckle images (1032 × 776 pixels) were obtained at 25 fps with an exposure time of 10 ms.

The LSCI system was controlled through a custom-made LabVIEW program provided by National Instruments (located in Austin, TX, USA). The analysis was performed using MATLAB software (version R2023b, provided by MathWorks, Inc., in Natick, MA, USA). To enhance the visualization of blood flow in real time with high resolution, a graphics processing unit (GPU) was integrated into the LSCI data processing framework. The GPU technology was developed by NVIDIA (based on Santa Clara, CA, USA) and utilizes parallel computing to significantly increase computing performance. In this study, we used a GeForce GTX 650 Ti GPU from NVIDIA.

A multichannel data acquisition system (Blackrock Microsystems, Salt Lake City, Utah, USA) was used to measure SSEPs. The signals were recorded through a head-stage amplifier with a gain of 2 and then filtered using a bandpass filter with a range of 0.5 Hz–7,500 Hz. The filtered signals were then analyzed.

#### Glycemic response to glucose administration

The glucose tolerance test measures the ability of the body to clear intraperitoneally injected glucose. It is used to detect disorders of glucose metabolism that may be associated with human diseases such as diabetes or metabolic syndrome. The animals were fasted for approximately 16 h, and fasting blood glucose levels were measured before the glucose solution was administered via intraperitoneal injection (IPI). Subsequently, blood glucose levels were measured at different time points over the next 2 h. Blood samples were collected from the tail vein, and glucose levels were measured with a glucometer before glucose loading (t = 0) and at 30, 60, and 120 min after glucose administration.^47^ The glucose tolerance of the rats in the different groups was evaluated by determining the area under the glucose curve (AUC glucose).

#### Insulin tolerance test (ITT)

It is recommended to assess insulin resistance to complement the *in vivo* analysis of glucose tolerance. Here, the ITT was selected, as it is a simple test and is technically very similar to the GTT in that it involves monitoring blood glucose levels over time but in response to insulin administration rather than a glucose load.^48^ Animals were fasted in clean cages from 8:00 a.m. to 2:00 p.m. (morning fast). The rats were intraperitoneally injected with recombinant human insulin (0.75 IU/kg body weight; Humulin R, Eli Lilly and Company, Indianapolis, IN, USA). Blood samples were obtained from the tail vein. Blood glucose levels were measured with a glucometer before loading (t = 0) and at 30, 60 and 120 min after insulin administration.^47^ The time course of the absolute glucose levels (mg/dL) is shown.

#### Animal preparation and surgery

A craniotomy was performed to create a window for the ECoG–LSCI. The rats were anesthetized with 2% isoflurane (Panion & BF Biotech, Inc., Taoyuan, Taiwan) in oxygen. A surgical drill was used to create a window covering ±4.0 mm anteroposterior (AP) and ±3.5 mm mediolateral (ML) to the bregma. For the ECoG recordings, two recording electrodes were placed in the region of the primary somatosensory cortex corresponding to the forelimb (S1FL) (AP = +1.0 mm, ML = +4 mm), and a reference electrode was placed posterior to the primary somatosensory cortex (ML = +4 mm from lambda). After surgery, the isoflurane concentration in the rats was reduced from 2% to 1.5% to monitor the response to electrical stimulation and KCl-induced CSD (Figure 2B).

#### Peripheral electrical stimulation

One stainless steel acupuncture needle was inserted in the palm of the left forelimb, and the other was inserted in the proximal forelimb muscle prior to electrical stimulation. A DS3 isolated current stimulator was used to apply 3 Hz rectangular pulse trains with a 0.2-millisecond width to the limb (Digitimer Ltd., Welwyn Garden City, Herforshire, UK). The highest current amplitude was 6 mA. Three blocks were combined to create a single-session stimulation paradigm. Each block consisted of 30 s of stimulation with ninety stimulation pulses interspersed with 30 s of rest (Figure 2B).

#### Monitoring CBF changes after electrical stimulation through LSCI

The rats were anesthetized using isoflurane, and a midline incision was made in the skin on the skull. To measure cerebral blood flow in rats, the RFLSI Pro+ laser speckle system (from RWD Life Sciences Co., Ltd., China) was used. Blood flow images were captured and analyzed to identify specific regions of interest (ROIs). The average blood flow indices in these ROIs were then calculated in real time.

#### KCl-induced CSD

After SSEPs were measured, CSD was induced with KCl. To determine CSD velocity, two points 3 mm apart were marked near the imaging viewport and scaled using a stereotaxic system. A hole was drilled in the lower right corner of the imaging field, and KCl was applied to induce CSD (as shown in Figure 2C). Recordings were taken 40 min after KCl application. LSCI images were used to determine the number of CSD events, the number of CSD occurrences, and the velocity of CSD. MATLAB was used to calculate the rCBF over time in a circular region of interest (ROI). The generated graph was used to determine the number of CSD events and the number of CSD occurrences.

The velocity of the CSD was calculated by comparing two regions of interest and dividing the distance between them by the time difference between the peaks in the two regions (X mm/Δt min). MATLAB was used to compute the velocity between two CSD points, accounting for the difference between the two points, the time difference between CSD in the two regions of interest, and the time difference between the peaks of cortical light spots. The wave crest direction of the CSD was from ROI 1 to area 2, as indicated by the arrow. During craniotomy, P1 (X, Y) and P2 (X, Y) were marked 3 mm apart on the left side of the skull, the coordinates were substituted into the formula $\sqrt{{\left(P1X-P2X\right)}^{2}+{\left(P2Y-P2Y\right)}^{2}}$ to calculate the distance between the two points, and two specific ROIs were identified in the image (circled along the direction of the CSD peak) to obtain the distance. The actual distance (mm) between the two ROIs was subsequently calculated and divided by the time difference to obtain the CSD speed (mm/s), which was subsequently converted to mm/min.

#### Analysis of LSCI data

To quantify rCBF changes at different time points in the cortical areas of the CSD, the normalized ratio of rCBF (rCBF_N_) was calculated as follows:

(Equation 1) $$r{CBF}_{N}\left(T_{n}\right)=\frac{R\left(T_{n}\right)}{R\left(T_{b}\right)}$$

where $R\left(T_{b}\right)$ is the baseline corresponding to the mean value of resting rCBF fluctuations before CSD and $R\left(T_{n}\right)$ is the mean value of resting rCBF fluctuations at the $n^{th}$ time window in the control and HFD/STZ groups.

The linear dependence of the resting rCBF fluctuations in the cortical area at each frequency was evaluated by means of the amplitude-squared coherence function given below:

The linear correlation of resting rCBF fluctuations in the cortical areas at each frequency was evaluated by the magnitude-squared coherence function given by the following equation:

(Equation 2) $${Coh}_{AM}^{2}\left(f\right)=\frac{{\left|P_{AM}\left(f\right)\right|}^{2}}{P_{AA}\left(f\right)P_{MM}\left(f\right)}$$

where *A* and *M* represent the rCBF signals in the cortex and $P_{AM}\left(f\right)$ represents the cross-power spectral density of the cortex. $P_{AA}\left(f\right)$ and $P_{MM}\left(f\right)$ represent the power spectral densities of the cortex. Welch’s overlapped averaged periodogram method was used to estimate the power spectral density.

In this study, the two rCBF signals were coherent in the frequency band (f) between 0.05 and 0.15 Hz, where the coherence values were greater than 0.5.^49^ Therefore, the phase of $P_{AM}\left(f\right)$ at frequencies between 0.05 and 0.15 Hz was used to indicate the relative lag between the coherent components, which was calculated to describe the temporal relationship of the rCBF between the cortical regions and is defined as follows:

(Equation 3) $$\emptyset \left(f\right)=\tan^{-1}\left[\frac{Im\left(P_{AM}\left(f\right)\right)}{Re\left(P_{AM}\left(f\right)\right)}\right]$$

where a positive phase difference (∅(f)) indicates the cortex as the blood in the cortical region is perfused through that cortical region, while a negative ∅(f) indicates that the rCBF in the cortex lags behind that in the cortical region as blood is perfused through another cortical region.^20^ Then, the time difference can be calculated by the following equation:

(Equation 4) $$\Delta t\left(f\right)=\frac{\emptyset \left(f\right)}{f}$$

#### Analysis of recorded ECoG data

The somatosensory-evoked potentials (SEEPs) induced by electrical stimulation of the forepaw were recorded and analyzed. MATLAB was used to perform offline analysis of the recorded SSEPs. The amplitudes of SSEPs were averaged across 90 individual scans, which yielded the mean SSEPs over a period of 0.2 ms after the stimulus pulse. The average SSEPs were then divided into the two most common SSEP components: P1 (first maximum voltage after stimulation) and N1 (minimum voltage). Changes in SSEP components, such as amplitude and peak latency, were used to assess changes in evoked responses to forepaw stimulation in the control and HFD/STZ groups.

#### Protein extraction

After monitoring KCl-induced CSD, the animals were sacrificed, and their brains were removed and placed on ice. The two hemispheres were then separated, and the hippocampus and cortex were dissected from each hemisphere and stored at −80°C. For protein extraction, hippocampal and cortical tissues were homogenized in RIPA buffer (Visual Protein, Taiwan) containing protease and phosphatase inhibitor cocktail tablets (Roche, Germany). The resulting homogenates were centrifuged at 13,000 rpm for 15 min at 4°C, and the protein concentrations were quantified using a BCA protein assay kit (Bio-Rad, USA). The proteins were subsequently stored at −80°C.

#### Western blotting

The samples were thawed on ice and then incubated in sample buffer (pH 6.8) supplemented with sodium dodecyl sulfate (SDS), β-mercaptoethanol, protease and phosphatase inhibitor cocktail, and bromophenol blue. The samples were then incubated with the mixture at 95°C for 10 min. A total of 20 μg of each protein sample was separated via gel electrophoresis on a 10% SDS‒polyacrylamide gel at 60 V for 3 h. The samples were then transferred to a PVDF membrane through wet transfer (30 V, 4°C overnight). The membrane was blocked using 5% nonfat milk in Tris-buffered saline (TBS) for 1 h at room temperature, followed by a 2-h incubation with primary antibodies against BDNF, NeuN, P-AKT, P-GSK3-β, and GFAP. All the primary antibodies were diluted in blocking buffer. The membrane was then washed with TBST before incubation with peroxidase-conjugated anti-rabbit or anti-mouse IgG secondary antibodies for 1 h. The immunoreactive bands were visualized using enhanced chemiluminescence (ECL) substrate. The signal intensity of each band was quantified using ImageJ software and compared with that of the β-actin band.

### Quantification and statistical analysis

All the values are expressed as the mean ± SEM. Multiple *t* tests were used for weight and blood glucose, unpaired *t* tests were used for FBG, AUC, amplitude and latency, Sidak multiple comparison tests were used for ITT, and Tukey multiple comparison tests were used for IPGTT. For blood changes, CSD velocity and CSD count were tested using Tukey’s multiple comparison test. The changes in CBF were compared with Sidak’s multiple comparison test. For Western blotting, an unpaired *t* test was used. Statistical analysis was performed using GraphPad Prism 8.0.1 (GraphPad Software, San Diego, CA, USA), and a significance level of *p* < 0.05 was used.
